# Oxidative Stress and Intervertebral Disc Degeneration: Pathophysiology, Signaling Pathway, and Therapy

**DOI:** 10.1155/2022/1984742

**Published:** 2022-10-10

**Authors:** Yanrun Li, Lu Chen, Yu Gao, Xuenong Zou, Fuxin Wei

**Affiliations:** ^1^Department of Orthopaedic Surgery, The Seventh Affiliated Hospital of Sun Yat-sen University, Shenzhen 518170, China; ^2^Department of Medical Ultrasonics, First Affiliated Hospital of Jinan University, Guangzhou 510070, China; ^3^Guangdong Provincial Key Laboratory of Orthopedics and Traumatology, Department of Spinal Surgery, The First Affiliated Hospital of Sun Yat-sen University, Guangzhou 510080, China

## Abstract

Intervertebral disc degeneration (IDD), characterized as decreased proteoglycan content, ossification of endplate, and decreased intervertebral height, is one of the major reasons of low back pain, which seriously affects the quality of life and also brings heavy economic burden. However, the mechanisms leading to IDD and its therapeutic targets have not been fully elucidated. Oxidative stress refers to the imbalance between oxidation and antioxidant systems, between too many products of reactive oxygen species (ROS) and the insufficient scavenging function. Excessive ROS can damage cell lipids, nucleic acids and proteins, which has been proved to be related to the development of a variety of diseases. In recent years, an increasing number of studies have reported that oxidative stress is involved in the pathological process of IDD. Excessive ROS can accelerate the IDD process via inducing the pathological activities, such as inflammation, apoptosis, and senescence. In this review, we focused on pathophysiology and molecular mechanisms of oxidative stress-induced IDD. Moreover, the present review also summarized the possible ideas for the future therapy strategies of oxidative stress-related IDD.

## 1. Introduction

Intervertebral disc degeneration (IDD) is an important pathologic process in intervertebral disc diseases and is usually associated with low back pain (LBP) [1]. It is estimated that about 80% of adults will experience low back pain in their lifetime, which can reduce their quality of life and even lead to disability, resulting in huge medical costs to our society [2]. Finding the mechanism and therapy for LBP will not only help the patients but also reduce the socioeconomic burden. Disc degeneration is a natural process of aging, which is characterized as decreased proteoglycan and water content, calcification of endplate, and decreased intervertebral height [3]. In addition to the degeneration caused by aging, there are many other factors that can exacerbate the degeneration. For example, excessive compression can promote disc degeneration [4]. While diagnostic imaging such as X-ray, Computed Tomography (CT), and magnetic resonance imaging (MRI) could be used to diagnose IDD in the clinic, MRI is the most effective imaging modality, and the classification scale of Pfirrmann (I-V) in T2-weighted images is often used to describe the grade of IDD [5]. However, a clear understanding of the pathogenesis of IDD is limited. Various pathologic processes involved in IDD include inflammation, extracellular matrix (ECM) degradation, autophagy, apoptosis, cell senescence, and nutrition deprivation. And a large number of studies have indicated that oxidative stress is involved in the abovementioned pathological processes [6–8]. Oxidative stress refers to the imbalance between oxidation and antioxidants caused by excessive reactive oxygen species (ROS) production and reduced antioxidant system function [9]. These evidences suggest that oxidative stress may be a new breakthrough in the treatment of IDD. This review provides an overview of the pathophysiology of IDD and mechanism of oxidative stress leading to IDD. Furthermore, the signaling pathway of oxidative stress-related IDD and the treatment for oxidative stress were summarized.

## 2. The Pathophysiology of IDD

Previous studies suggest that the etiology of IDD is multifactorial and affected by genetics [10], gender [11], aging [12], high mechanical stress [13], and disc dystrophy [14, 15]. The anatomically intact intervertebral disc (IVD), especially the nucleus pulposus (NP), is commonly considered an avascular organ except for the outer annulus fibrosus (AF) layer. Nutrients reach the IVD predominantly by diffusion through the vertebral endplate, so the NP has precarious nutrition [16]. Endplate has been demonstrated to be remodeled during aging, undergoing gradual ossification with elevated osteoclasts number and increased porosity [17–20], thereby leading to disc degeneration [21, 22]. In our previous studies, we successfully established an intervertebral disc degeneration model in rhesus monkeys [23, 24] by injecting pingyangmycin, an antiendothelial drug, into the subendplate region to block the blood sinus in the endplate. This study supported the dystrophy disorder hypothesis. In addition, mechanical loading such as torsion, compression, and bending can also cause damage to the IVD structure, including AF tears and NP herniation, and further accelerate IDD by changing the cell homeostasis of the IVD [25]. Besides, the degenerated IVD is more vulnerable to mechanical loading. IDD is also closely related to age. With the increase of age, senescence cells caused by various in vivo and in vitro factors gradually increase in IVD. Due to the lack of blood vessels in IVD itself, the clearance of senescence cells is slow, which makes them further accumulate, showing pathological processes such as inflammation and catabolic phenotypes and accelerating IDD [26]. Under normal conditions, the NP is a gel-like, highly hydrated tissue composed of ECM rich in type II collagen and proteoglycan. Healthy AF is a highly organized fibrous structure composed of type I collagen fiber [27, 28]. However, in IDD, catabolism is enhanced and anabolism is weakened, proteoglycans and type II collagen were gradually replaced by disorder type I collagen, and water in IVD also decreases, which destroys the composition and function of IVD [29]. Except for the pathogenesis mentioned above, there are many other causes of IDD. This review will focus on the role of oxidative stress in IDD.

## 3. Oxidative Stress

**Figure 1:**
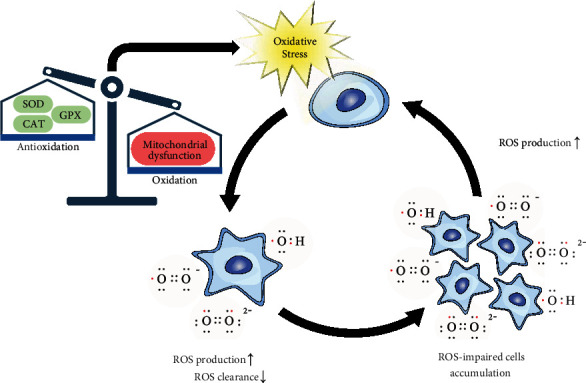
The vicious positive feedback loop in oxidative stress. When the balance between oxidation and antioxidation is broken, the body will undergo oxidative stress. On the one hand, ROS can impair cells under oxidative stress. Impaired cells, on the other hand, produce more ROS, exacerbating oxidative stress. ROS: reactive oxygen species; SOD: superoxide dismutase; CAT: catalase; GPX: glutathione peroxidase.

ROS are generated as byproducts of aerobic metabolism, which have stronger reactivity than molecular oxygen [30], mainly including oxygen singlet, superoxide anion (O_2_^·–^), hydrogen peroxide (H_2_O_2_), and hydroxyl radical (OH^·–^) [31]. The high reactivity of ROS allows it to damage the nucleic acids, proteins, and lipids, so an excess of ROS is generally considered to be harmful to health. The vast majority of ROS are generated by electron transport in the respiratory chain on the inner membrane of mitochondria [31]. Under normal conditions, through the respiratory chain, electrons from metabolite are transferred to molecular oxygen via complexes (I to IV) to form water, and complex IV can ensure complete reduction of molecular oxygen, along with ATP generation. However, some electrons escape from the respiratory chain during this process, resulting in incomplete reduction of molecular oxygen, which means the generation of ROS. It is generally believed that complexes I and III of the respiratory chain are the main sites of ROS generation [31, 32]. Of course, in addition to generating ROS, the human body also has antioxidant systems for scavenging ROS. Antioxidant systems that counteract ROS can be divided into enzyme and non-enzyme systems. Enzyme systems include superoxide dismutase (SOD), catalase (CAT), glutathione peroxidase (GPX), and nonenzyme systems mainly include reduced glutathione (GSH) and vitamin C/E [33]. Under the normal state, the body is in the homeostasis of oxidation and anti-oxidation, but there are also some factors that can break this balance (Figure 1). For example, mitochondrial dysfunction is a common pathologic condition in IDD, which is known as loss of mitochondrial mass, defection of respiratory chain, opening of mitochondrial permeability transition pore, and decline in mitochondrial membrane potential [34]. More electrons leak out of the respiratory chain in the dysfunctional mitochondrion, leading to an overproduction of ROS [34]. At the same time, the function of antioxidant enzymes in intervertebral disc was inhibited, and the ROS clearance rate decreases [35]. It was found that the higher the degeneration degree of intervertebral disc, the lower the SOD activity in rat [36]. When ROS generation is excessive and the function of antioxidant system decreases, the body will be in a state of imbalance between oxidation and antioxidant, which is called oxidative stress [30]. Further leading to cell impairment and abnormal function. The accumulation of these ROS-impaired cells will further accelerate the ROS generation and build a vicious cycle, which will eventually induce relative disease (Figure 1).

## 4. Cellular and Animal Models of Oxidative Stress

At present, cell model and animal model are the main methods to study oxidative stress and antioxidant in vitro and in vivo, respectively. Cell models of oxidative stress can be formed by stimulating cells with special stressors. These stressors can damage deoxyribonucleic acid (DNA), ribonucleic acid (RNA), protein, lipid, and other intracellular components, which play a toxic role and promote oxidative stress. [37]. Stressors can be divided into chemical stressors and physical stressors. There are many kinds of chemical stressors, among which H_2_O_2_ is the most common one. As a kind of ROS, it can inhibit cell proliferation, cause oxidative damage of intracellular macromolecules, and eventually lead to serious consequences such as cell senescence, death, and mutation. Therefore, H_2_O_2_-induced oxidative stress cell model is widely used to explore the mechanism of cell damage mediated by free radicals and the protection and repair mechanism of antioxidant on oxidative damage [38]. Interleukin-1*β* (IL-1*β*) can induce NP degeneration by inducing apoptosis, inflammation, and oxidative stress of NP cells. Li et al. established a NP cell degeneration model using IL-1*β* to study the roles of p16 regulating the proliferation and senescence of NP cells by affecting oxidative stress and cell cycle and the protection effect of rapamycin on it [7]. Some chemicals with neurotoxicity can also be applied into the study of oxidative stress related diseases. As a metabolite of neurotoxin 1-methyl-4-phenyl-1,2,5,6-tetrahydropyridine (MPTP) in vivo, 1-methyl-4-phenylpyridinium (MPP (+)) can inhibit mitochondrial complex I and cause excessive production of ROS, resulting in intracellular oxidative stress and apoptosis. It is often used to establish cell models of Parkinson's disease [39]. Some chemicals can be used as free radical generators. 2,2-Azobis(2-amidinopropane)dihydrochloride (AAPH) is a water-soluble azo small molecule that can induce the generation of oxygen free radical and lead to various pathological changes through oxidative damage of cells. Therefore, AAPH, as a source of free radical generation, is often used to study the antioxidant activity of red blood cells, plasma, and whole blood [40]. In addition, drugs can also be used to establish oxidative stress cell models. For example, doxorubicin (DOX) is an effective chemotherapy drug, but it can cause a variety of cardiotoxicity, such as tachycardia, arrhythmia, and hypotension. Oxidative stress is the key factor of DOX induced myocardial injury. Because DOX can produce a large number of superoxide anion radicals, resulting in mitochondrial dysfunction and cell damage [41]. Physical stressors mainly include ultraviolet radiation and air particles. It comes from the external environment and will cause oxidative damage to the body. Hou et al. exposed mouse embryonic fibroblasts to an 1800 MHz GSM-talk mode electromagnetic radiation (EMR) intermittently to evaluate its oxidative damage to cells [42]. Oxidative stress induced by PM2.5 is an important molecular mechanism of its toxic effect. Deng et al. established a cell model with PM2.5 to explore the antioxidant defense mechanism related to PM2.5 in cells [43]. In addition to cell models, researchers have also used animal models to study oxidative stress. In IDD-related oxidative stress, researchers mostly build animal models through traditional methods such as needle puncture, tail suspension, and ovariectomy and then study oxidative stress under these IDD models [44–46]. At present, there is still a lack of specific oxidative stress-related IDD animal models, which also makes the research in this field mainly focus on cell models. Artificial selection of disease-related stressors and simulation of oxidative stress can reflect the different mechanisms of antioxidant action in the pathological state of the body. Therefore, the selection of stressors should consider the research purpose. To study the oxidative damage effect of a certain substance, the substance can be selected as the stressors to establish the cell model. To evaluate the antioxidant activity of antioxidants, stressors should be selected according to the action target and mechanism of antioxidants or directly choose the commonly used stress methods.

## 5. The Mechanisms of Oxidative Stress-Related IDD

### 5.1. Inflammation

**Figure 2:**
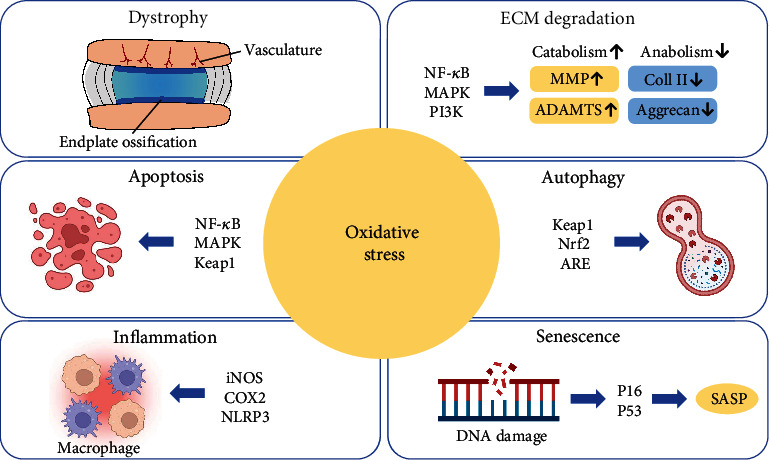
The mechanisms of oxidative stress-related intervertebral disc degeneration. Excessive ROS are produced in a state of oxidative stress; these ROS can further induce intervertebral disc degeneration through various mechanisms. ROS can cause the ossification of cartilage endplate and hinder the transportation of nutrition and metabolite in NP. ROS can also cause ECM degradation by converting anabolism to catabolism. ROS-induced inflammation, apoptosis, and autophagy can disturb the cell function and reduce the cell number in NP via multiple pathways. Moreover, DNA damage caused by ROS can activate p53 and p16, then lead to cell senescence. ROS: reactive oxygen species; ECM: extracellular matrix; NP: nucleus pulposus; MMP: matrix metalloproteinase; ADAMTS: a disintegrin and metalloproteinase with thrombospondin motifs; Coll II: type II collagen.

Inflammation is a defensive response of the body to stimuli. Under normal circumstances, inflammation is beneficial and is the body's automatic defensive response; however, sometimes inflammation is also harmful, such as attacks on the body's own tissues. There are various inflammatory factors that play a role in the process of IDD, such as IL-1*α*, IL-1*β*, IL-6, and tumor necrosis factor *α* (TNF-*α*). Patients with IDD often have chronic low back pain and may have symptoms of nerve root pain, which may be caused by inflammation that stimulates the nerve endings of the sinus vertebral [27]. Through single-cell sequencing technology, studies have found that inflammation-related immune cells infiltrate in IDD, including Tregs and macrophages [47] (Figure 2). At the same time, inflammation in the IVD can also lead to catabolism, cell senescence, and apoptosis, which can accelerate the pathological process of IDD [48–50]. Oxidative stress is involved in the process of IDD, and ROS is also one of the important factors that induce inflammation. Studies have found that H_2_O_2_ can promote the expression of inflammatory gene including inducible nitric oxide synthase (iNOS), cyclooxygenase 2 (COX2), and IL-6 in NP cells [51] (Figure 2). Besides, H_2_O_2_ can increase the inflammatory response by activating nucleotide-binding oligomerization domain, leucine-rich repeat, and pyrin domain-containing protein 3 (NLRP3) inflammasome [51]. Not only oxidative stress can cause inflammation, inflammation can also in turn enhance oxidative stress. Mitochondria stimulated by tumor necrosis factor (TNF) can increase ROS, leading to apoptosis and necroptosis, while ROS can also promote the generation of TNF, forming a positive feedback loop between them [52]. This loop is also found during the process of disc degeneration [53]. This indicates that during the IDD process, inflammation and oxidative stress promote each other, finally leading to a vicious circle of IDD.

### 5.2. Dystrophy

The intervertebral disc is the largest avascular tissue in the human body. The absorption of nutrients and the discharge of metabolic waste from the intervertebral disc are completed by the capillary network of adjacent vertebral body [54]. Nutrients move from the capillaries to the intervertebral disc cells mainly by diffusion through the cartilage endplate and the intervertebral disc matrix, while the metabolites leave the intervertebral disc in the opposite direction [54]. In the process of IDD, the lesions of the cartilage endplate will block the above-mentioned pathways, which will further lead to the impaired cellular activity and viability (Figure 2). Some studies have confirmed that ROS can also affect the function of cartilage endplate cells during IDD. In mouse cartilage endplate tissue, TNF-*α* are capable to increase the level of intracellular ROS and further lead to osteogenic differentiation of the cartilage endplate stem cells [55]. The calcification of endplate will hinder the normal metabolism of the IVD [54]. In human cartilage endplate cells, H_2_O_2_ can promoted ROS generation, mitochondrial dysfunction, and apoptosis [56]. The above studies suggest that ROS can induce dysfunction and phenotype changes of cartilage endplates, which may lead to NP cell nutritional metabolism disorders, thereby further aggravating the progress of IDD.

### 5.3. ECM Metabolism

Only by maintaining a healthy ECM structure and composition can the intervertebral disc function normally. However, in IDD, the metabolism of ECM is often disturbed [28]. In IDD, the catabolism of the ECM in the intervertebral disc gradually increases, while the anabolism decreases [29]. At the same time, proteoglycans and type II collagen were gradually replaced by type I collagen [29]. Oxidative stress is involved in the switch of metabolic state and composition. Tert-butyl hydroperoxide (TBHP) can downregulate anabolism markers (type II collagen and aggrecan mRNA levels) and upregulate catabolism markers including matrix metalloproteinase- (MMP-) 3, MMP-13, a disintegrin and metalloproteinase with thrombospondin motifs (ADAMTS) 4, and ADAMTS-5 mRNA levels in human NP cell [57] (Figure 2). Besides, in AF cells, H_2_O_2_ treatment promote catabolism-related mRNA expression such as TNF-*α*, MMP-3, and COX-2 [53]. In short, oxidative stress can undermine the metabolic balance of ECM, leading to changes in the composition of intervertebral disc.

### 5.4. Apoptosis

Apoptosis refers to the orderly and autonomous death of cells controlled by genes in order to maintain the homeostasis. Moderate apoptosis is necessary to maintain the tissue function, but excessive apoptosis often leads to structural and functional abnormalities. In the intervertebral disc cells, various stimulating factors activate the apoptotic pathways including the exogenous death receptor pathway, endogenous mitochondrial pathway, and endoplasmic reticulum stress (ERS) pathway; induce cell death; and ultimately lead to the occurrence of IDD [58, 59]. Oxidative stress also plays an important role in IDD induced by apoptosis. In rat NP cells, H_2_O_2_ can decrease the mitochondrial membrane potential and enhance apoptosis by activating the mitochondrial pathway, which is characterized as nuclear condensation. At the same time, impaired mitochondria can also cause overproduction of ROS [60]. Besides, H_2_O_2_ increase the permeability of lysosomal membrane in the NP cells and further induce apoptosis through the mitochondrial pathway [61]. Studies have also shown that ROS can induce apoptosis through other pathway like the death receptor [62], but in IDD, ROS mainly induce apoptosis through mitochondrial pathways [34]. The apoptosis induced by ROS can impair the survival of cells and lead to the decline of cell number, which hinder the metabolism and function of disc.

### 5.5. Autophagy

Autophagy is the process of encapsulating cell's own cytoplasmic proteins or organelles into vesicles, fusing with lysosomes to form autophagic lysosomes, and degrading the contents it encapsulates, thereby fulfilling the metabolic needs of the cell itself. Autophagy can be seen in both physiological and pathological processes of the body. Autophagy is in a dynamic balance, both too strong and too weak autophagy will have an adverse effect on cells [63]. Autophagy is associated with IDD; Pfirrmann grade III IDD has increased number of autophagic lysosomes, which lead to the decomposition of degenerative substances [64]. Oxidative stress is also involved in autophagy-induced IDD. Double-membrane enclosed autophagosomes are significantly increased in NP cells treated with H_2_O_2._ Moreover, H_2_O_2_ can also increase the expression of autophagic markers, which may further accelerate cell death [6]. However, in addition to the role of autophagy in promoting the process of IDD, autophagy can also protect the intervertebral disc cells from damage caused by oxidative stress. In autophagy-deficient cells, H_2_O_2_-induced oxidative stress aggravates the production of ROS, which indicate the protective effect of autophagy on oxidative stress damage [6]. Moreover, the antioxidant Keap1-Nrf2-ARE pathway can restore autophagic flux disturbances caused by TBHP and further alleviate apoptosis [65]. Therefore, autophagy is a double-edged sword in IDD caused by oxidative stress, excessive autophagy can boost the death of cell, but appropriate autophagy can increase the survival of cell.

### 5.6. Senescence

Cell senescence refers to the irreversible cell-cycle arrest that occurs under various stimuli including telomere shortening and DNA damage [66]. Roberts et al. have provided evidence that the level of senescence is increased in IDD [67]. Senescence can weaken the proliferation of intervertebral disc cells, so as the cells die, the number of functional cells in the intervertebral disc gradually decreases. Moreover, the senescence-associated secretory phenotype (SASP) of aging intervertebral disc cells is transformed into catabolic and inflammatory phenotype, which lead to the acceleration of IDD [68, 69]. In terms of mechanism, study have demonstrated that the effects of senescence in the intervertebral disc are mainly regulated by p16 and p53 [66] (Figure 2). Oxidative stress is usually one of the critical factors that trigger cell senescence [70]. The deficiency of p16 can attenuate IDD by decreasing oxidative stress, which indicate that oxidative stress plays an important role in the cell senescence of IDD [7]. Besides, ROS can cause DNA damage and further induce the activation of p53-p21-Rb and p16-Rb pathways to intensify cell senescence in NP [71]. Future research needs to clarify the relationship between oxidative stress and cellular senescence, which may provide a new target for the treatment of IDD.

## 6. Signaling Pathways Involved in Oxidative Stress-Related IDD

### 6.1. NF-*κ*B

The nuclear factor NF-*κ*B family consists of five members: RelA (p65), RelB, c-Rel, and precursor proteins NF-*κ*B1 (p105) and NF-*κ*B2 (p100) [72]. NF-*κ*B plays a key role in cellular inflammation and immune response, and meanwhile, it is also involved in many other cellular regulatory processes, including proliferation, differentiation, autophagy, and apoptosis [73]. So, the dysfunction of NF-*κ*B is associated with a variety of diseases like diabetes, arthritis, and cancer [74–76]. The activation of NF-*κ*B pathway is mainly related to the phosphorylation of I*κ*B-kinase (IKK) *β* and the phosphorylation and degradation of I*κ*B*α* [77]. Some studies believe that ROS also play a role in the activation of NF-*κ*B pathway. Exogenous addition of H_2_O_2_ can cause the phosphorylation and degeneration of I*κ*B*α*, thereby leading to the activation of NF-*κ*B pathway [78]. Besides, ROS can influence the activity of IKK*β* by affecting its S-glutathionylation, which further regulates the NF-*κ*B pathway [79]. Similarly, NF-*κ*B pathways are involved in oxidative stress-induced IDD (Table 1). In the study of Feng et al., rat NP cells were cultured under 20% O_2_, in which condition ROS was overproduced. The excessive ROS induce catabolic and proinflammatory phenotype of NP cells via NF-*κ*B pathways [71]. The in vitro and in vivo experiments from Zhao et al. indicated that increased activity of NF-*κ*B pathway might had been associated with the ROS-related IDD observed in mouse NP cells and NP tissue [80]. Similarly, in the rat annulus fibrosus (AF) cell, ROS may also induce IDD by activating NF-*κ*B pathways [81]. Moreover, Nerlich et al. found that oxidative stress was positively correlated with the activation degree of NF-*κ*B pathway and the degeneration degree of disc in human intervertebral disc tissues [82]. In addition to NP and AF, lesions of the cartilage endplate also result in IDD. Han et al. cultured cartilage endplate cells isolated from human lumbar intervertebral discs at different concentrations of H_2_O_2_ for different periods of time. H_2_O_2_-induced oxidative stress increases apoptosis of cartilage endplate cells through the p65 pathway in a dose-dependent manner, which eventually lead to calcification and IDD [83].

### 6.2. MAPK

The family of MAPK (mitogen-activated protein kinase) consists of extracellular signal-regulated kinase (ERK), p38, and c-Jun NH_2_-terminal kinase (JNK). Each MAPK signaling contains at least three parts: MAPK kinase kinase (MAP3K), MAPK kinase (MAP2K), and MAPK [84]. MAPK regulates cell functions mainly through the phosphorylation of downstream substrates [85]. These signaling pathways get involved in various cellular activities including cell survival, death, proliferation, differentiation, inflammation, and immunity [84], which is related to the development of multiple diseases, such as cancer and degenerative diseases [84]. In the meantime, these diseases are also linked to oxidative stress [86]. Some studies indicate oxidative stress can play as a pathogenic mechanism in these diseases through the MAPK signaling pathway (Table 1). H_2_O_2_ can increase the level of intracellular ROS and activate ERK, p38, and JNK signaling pathways in human NP cell [87]. Besides, in human cartilage endplate cells, the apoptosis can be accelerated by the H_2_O_2_-induced oxidative stress via the p38/ERK pathway and eventually cause the calcification of cartilage endplate [83]. Recently, Zhang et al. also provide evidence that downregulation of p38/MAPK pathway can alleviate the apoptosis of rat NP cells and protect extracellular matrix from degradation, which indicates that the p38/MAPK pathway can exaggerate the apoptosis of IDD [88]. In addition to apoptosis, ROS can cause DNA damage and further induce the activation of p53-p21-Rb and p16-Rb pathways via ERK pathway to promote NP cell senescence [71]. Moreover, ROS can also accelerate catabolic and pro-inflammatory phenotypes of NP cell through the MAPK pathway [71]. Xu et al. found that H_2_O_2_-induced ROS overproduction promotes the expression of MMP3, MMP9, and MMP13 via p38/MAPK-mediated phosphorylation in human NP cells. Furthermore, this effect of ROS can be abolished by the administration of the inhibitors of p38/MAPK [89]. The activated MAPK signal pathway in IVD can increase a variety of inflammation factors such as TNF- *α* and IL-6, which leads to IDD through the abovementioned inflammation mechanism [53, 90]. Besides, a positive feedback loop was established between the over activation of MAPK and TNF-*α* in IDD. On the one hand, ROS can promote the production of TNF-*α* via p38 pathway. On the other hand, TNF-*α* can cause excessive ROS production by activating MAPK [53].

### 6.3. Keap1-Nrf2-ARE

The Keap1-Nrf2-ARE pathway consists of Kelch-like ECH-associated protein 1 (Keap1), nuclear factor erythroid 2-related factor 2 (Nrf2), and antioxidant response elements (ARE), which play as the defense system for oxidative stress [91]. In physiological condition, Keap1 can degrade Nrf2 by ubiquitination. However, when the balance of ROS production and clearance is destroyed, Keap1 will be inactivated, which leads to the obstruction of Nrf2 clearance and finally leads to the excessive accumulation and activation of Nrf2. Nrf2 then translocates into the nucleus and targets ARE to regulate the expression of genes related to antioxidant stress [91]. Nrf2 is also activated in disc degeneration, suggesting an interaction between IDD and oxidative stress. The mRNA expression of Nrf2 was negatively correlated with the Pfirrmann grades in NP tissues from IDD patients [6]. More Nrf2 positive NP cells are also detected in NP tissues with high degree of degeneration [6]. Besides, the development of IDD can be accelerated by knocking out Nrf2 [6]. Elevated ROS levels can increase both autophagy and Nrf2 in human NP cells, which indicate the correlation between autophagy and Nrf2 in oxidative stress-related IDD [92]. Tang et al. found that H_2_O_2_-induced excessive accumulation of Nrf2 can protect cell from oxidative stress through Keap1-Nrf2-p62 autophagic pathway [6]. Moreover, the upregulation of Nrf2/Sirt3 pathway can alleviate the apoptosis and mitochondrial dysfunction in TBHP-induced rat IDD NP cell, and the upregulation of Nrf2/Sirt3 pathway also restored the disturbed autophagy in this model [65]. In addition to NP cell, Nrf2 plays an important role in endplate chondrocytes. Knockdown of Nrf2 increased ROS-related harmful effects, including mitochondrial dysfunction and apoptosis [56]. Zuo et al. provide evidence that rapamycin-induced autophagy can enhance the Nrf2/Keap1 pathway in endplate chondrocytes and increase the production of antioxidant, then counteracting the damage from ROS, reducing the osteogenic differentiation of chondrocytes, and alleviating cell senescence [55]. In summary, the Keap1-Nrf2-ARE pathway can restore autophagy and antioxidation to resist IDD induced by oxidative stress (Table 1).

### 6.4. PI3K-Akt

The PI3K- (phosphoinositide-3-kinase-) Akt (protein kinase B) pathway plays an important role in various critical cellular functions, such as protein synthesis and cell survival [93, 94]. On the one hand, ROS can directly activate PI3K and promote its downstream signal transduction. On the other hand, ROS are capable to inactivate phosphatase and tensin homolog (PTEN), which can inhibit the activation of Akt through its negative regulation of PIP3 synthesis [95]. PI3K and Akt are significantly reduced in human NP cells cultured with H_2_O_2_ [96]. In IDD patients, the activation of PI3K/Akt can increase the proliferation of NP cells, whereas the inhibition of PI3K/Akt can reverse this effect [97]. Besides, Akt may get involved in the cell senescence induced by H_2_O_2_ in rat NP cell via the silent information regulator 1 (Sirt1) pathway [98]. Moreover, PI3K/Akt can regulate the ROS production and the activity of MMP [99].

## 7. Therapy Target for Oxidative Stress-Related IDD

### 7.1. Natural Origin

Curcumin (CUR) extracted from the curcuma longa is an active polyphenol [100]. It has been reported to have the function of inducing autophagy [101]. The disturbed autophagy is usually an important pathogenic factor in oxidative stress-induced IDD [57]. In human NP cells exposed to TBHP, CUR can restore the defective autophagic flux via AMP-activated protein kinase (AMPK)/mechanistic target of rapamycin (mTOR) pathway [57]. CUR may achieve this effect by alleviating impaired autophagosome-lysosome fusion and lysosomal function [57]. Besides, CUR can protect the NP cells from TBHP-induced apoptosis, senescence, and ECM degradation [57]. In addition to CUR, berberine, an isoquinoline alkaloid isolated from coptidis rhizome and cortex phellodendri, is also involved in the regulation of autophagy. Berberine can reduce H_2_O_2_-induced apoptosis through the modulation of endoplasmic reticulum stress and autophagy; this effect may be achieved through c-Jun NH2-terminal kinase (JNK) pathway and deregulation of Ca^2+^ [102]. Danshen (Salvia miltiorrhiza) is a traditional Chinese medicine. Due to its function of promoting blood circulation and removing blood stasis, Danshen is widely used in the treatment of cardiovascular diseases. In addition to the function above, Danshen also has the ability of decreasing ROS. Qin et al. found that 4 weeks gavage of Danshen can increase the level of GSH and SOD2 and decrease malondialdehyde (MDA) in IDD rats, which reflects the antioxidant function of Danshen [44]. Recently, Dai et al. also provide evidence that salvianolic acid B (extracted from Danshen) can restore the abnormal ROS, GSH, SOD2, and MDA levels in H_2_O_2_-induced oxidative stress [103]. This antioxidant effect of salvianolic acid B may be mediated by the Janus kinase 2 (JAK2)/signal transducer and activator of transcription 3 (STAT3) pathway [103]. Moreover, acacetin is another important compound derived from Agastache rugosa and is also an antioxidant traditional Chinese herb. Acacetin is able to reduce the THBP-Induced overproduction of ROS and increase the expression of antioxidant proteins including heme oxygenase-1 (HO-1) and quinone 1 (NQO1) [104]. Besides, acacetin protects rat NP cell from inflammation and ECM degradation caused by TBHP [104]. They also found that the antioxidant Nrf2 pathway may get involved in the function of acacetin in IDD cell [104]. Above all, herbs, such as Danshen and Agastache rugosa, can play an important role in the therapy of IDD (Table 2).

### 7.2. Chemical Medicine

Metformin is the first-line medicine for type 2 diabetes, which is widely used around the world. In addition to its function of decreasing blood glucose, metformin also can alleviate oxidative stress and enhance autophagy [105]. In rat NP cells, metformin can effectively restore the defective autophagy flux caused by TBHP and further protect the NP cells from apoptosis [105]. Besides, metformin inhibits TBHP-induced cells' senescence and regulates the expression of degeneration-related genes via autophagy, which was further confirmed by the autophagy inhibitor [105]. For instance, metformin can reverse the dysregulation of collagen-II, aggrecan, MMP-3, and ADAMTS-5 induced by TBHP [105]. Pioglitazone, another pharmacologic treatment for diabetes, also can reduce the damage induced by oxidative stress. In human NP cells, Hu et al. found that pioglitazone can significantly reduce ROS production and MDA caused by compression [63]. Moreover, aspirin can decrease the LPS-induced ROS expression in rat NP cells and activate the Nrf2 pathway [106]. Using the PI3K inhibitor, Qi et al. indicate that dexmedetomidine can suppress IL-1*β*-induced oxidative stress via the PI3K/Akt pathway [107]. These studies proved that chemical medicines are capable to alleviate IDD through multiple pathways (Table 2).

### 7.3. Biomaterials

Except for medicine, biomaterials are also used in the treatment of IDD. In cartilage endplate stem cells, rapamycin enhances the expression of antioxidant enzymes and decreases the production of ROS via autophagy [55]. Bai et al. construct an in situ formed ROS-scavenging scaffold loaded with rapamycin, which could be utilized to inhibit the production of ROS as well as achieve the ROS-triggered rapamycin release at the oxidative stress site [108]. This rapamycin-loaded ROS scavenging hydrogel was locally injected into the degenerative intervertebral discs of rats. In addition to removing ROS and releasing rapamycin, the hydrogel can also accelerate polarization of macrophages to M2-type, which is involved in the anti-inflammatory and tissue repairment [108]. Due to its nanostructure, long-lasting activity, and cell membrane penetration, fullerene is more powerful than traditional antioxidants [109]. Yang et al. found that nanofullerol decreases the ROS of H_2_O_2_-cultured human NP cells [110]. Besides, nanofullerol can reverse the H_2_O_2_-induced dysregulation of COL I, COL II, aggrecan, MMP3, MMP9, and ADAMTS5, which indicates nanofullerol can against the matrix destruction caused by oxidative stress [110]. Liu et al. also provide evidence that nanofullerol can suppress the production of ROS as well as inhibit adipogenesis in vertebral bone marrow stromal cells, which may reduce vertebral fatty marrow deposition and inflammatory responses during disc degeneration [111]. Recently, Yu et al. synthesize an amphiphilic copolymer, which could self-assemble into a nanosized micelle and load lipophilic kartogenin, as a single complex (PAKM) [112]. PAKM can improve cell viability in adipose-derived stem cells treated by H_2_O_2_ and alleviate the oxidative stress by upregulating SOD expression [112]. Moreover, in adipose-derived stem cells, PAKM is able to protect disc from matrix degeneration and promote NP differentiation in the condition of oxidative stress [112]. Biomaterials may provide multiple possibilities for IDD treatment in the future.

### 7.4. Stem Cell Therapy

In addition to the above treatment methods, stem cell therapy for IDD has attracted more and more attention in recent years. Some studies have found that stem cell transplantation can effectively alleviate IDD [113] (Table 2). One of the important mechanisms of stem cell therapy for IDD is antioxidant stress. Chen et al. found that mesenchymal stem cell- (MSC-) derived exosomes can inhibit the production of ROS and ameliorate mitochondrial dysfunction [51]. Hu et al. also found that MSC-derived exosomes can reduce ROS and MDA levels in NP oxidative stress caused by compression [114]. Except for MSC, cartilage endplate stem cell-derived exosomes can inhibit TBHP-induced NP cell apoptosis by activating the PI3K/Akt signaling pathway and promoting autophagy [115]. Although stem cell therapy can alleviate IDD by inhibiting oxidative stress, the oxidative stress microenvironment will also deteriorate the survival of stem cells. Therefore, some studies began to focus on how to make stem cells withstand the damage of local oxidative stress. Studies have shown that some medicine like cyclosporine A and 1,25(OH)_2_D_3_ can alleviate the apoptosis of MSC caused by oxidative stress and promote the survival of MSC [116, 117]. At present, the abovementioned natural origin, chemical medicine, biomaterials, and stem cell therapy are still in the stage of cell and animal experiments. In the future, clinical trials are imperative to further explore the effects of these treatments for oxidative stress-related IDD.

## 8. Conclusion

In this review, we make a summary about the mechanisms by which oxidative stress induces IDD, including inflammation, ECM degradation, autophagy, apoptosis, cell senescence, and dystrophy. In addition, multiple signaling pathways that are activated by oxidative stress or participated in the pathological process are also reviewed. What is more, we also summarize the IDD therapy strategies targeting oxidative stress from natural origin, chemical medicine, biomaterials, and stem cell therapy. However, the mechanism of oxidative stress-induced IDD is complex and has not been fully elucidated, existing treatments for antioxidant stress are insufficient for protecting the intervertebral disc from degeneration, and clinical trials are also lacking. Future studies need to further explore the signaling pathways through which ROS damage occurs in IVD cells and provide specific therapeutic targets for oxidative stress-related IDD.

## Figures and Tables

**Table 1:** Signaling pathways involved in oxidative stress-related intervertebral disc degeneration.

Signaling pathway	Experimental models (stimuli)	Cellular processes regulated by the signaling pathway	Reference
NF-*κ*B	Rat NP cell (20% O_2_)	Catabolism↑, inflammatory↑	[71]
	Rat AF cell (TNF-*α*)	Cell senescence↑	[81]
	Mice NP cell (TNF-*α*)	ROS↑	[80]
	Human cartilage endplate cell (H_2_O_2_)	Apoptosis↑	[83]

MAPK	Rat NP cell (20% O_2_)	Cell senescence↑, catabolism↑, inflammatory↑	[71]
	Rat NP cell (TBHP)	Apoptosis↑	[88]
	Rat AF cell (H_2_O_2_, BSO and TNF-*α*)	Anabolism↓, catabolism↑	[53]
	Human NP cell (H_2_O_2_)	Catabolism↑	[89]
	Human NP cell (H_2_O_2_)	Cell senescence↑, catabolism↑	[87]
	Human cartilage endplate cell (H_2_O_2_)	Apoptosis↑	[83]

Keap1–Nrf2–ARE	Mice endplate chondrocytes (TNF-*α*) Rat NP cell (TBHP)	ROS↓, cell senescence↓, osteogenic differentiation↓ Apoptosis↓, mitochondrial dysfunction↓, autophagy↑	[55] [65]
	Human NP cell (H_2_O_2_)	Autophagy↑	[6]
	Human endplate chondrocytes (H_2_O_2_)	Apoptosis↓, mitochondrial dysfunction↓	[56]

PI3K-Akt	Rat NP cell (H_2_O_2_)	Cell senescence↑, inflammatory↑	[98]
	Rat NPMSC cell (H_2_O_2_)	ROS↓, catabolism↓, mitochondrial dysfunction↓	[99]
	Human NP cell (H_2_O_2_)	Survival of cells↑	[97]

NP: nucleus pulposus; AF: annulus fibrosus; ROS: reactive oxygen species; CST: cortistatin; TBHP: tert-butyl hydroperoxide; BSO: buthionine sulfoximine; NPMSC: nucleus pulposus-derived mesenchymal stem cells.

**Table 2:** Therapy target for oxidative stress-related intervertebral disc degeneration.

Classification	Antioxidant (dose & time)	Experimental models (stimuli)	Therapeutic effects	Reference
Natural origin	Curcumin	Human NP cells (TBHP)	ROS↓, autophagy↑, apoptosis↓, cell senescence↓, catabolism↓	[57]
	Berberine	Human NP cells (H_2_O_2_)	ER stress↓, autophagy↓, apoptosis↓	[102]
	Danshen	Rats (needle puncture)	ROS↓, inflammatory↓, catabolism↓	[44]
	Salvianolic acid B	Rats (needle puncture)	ROS↓, proliferation↑, apoptosis↓	[103]
	Acacetin	Rat NP cells (TBHP)	ROS↓, inflammatory↓, catabolism↓	[104]

Chemical medicine	Metformin	Rat NP cells (TBHP)	Apoptosis↓, senescence↓, catabolism↓, anabolism↑	[105]
	Pioglitazone	Human NP-MSC cells (compression)	ROS↓, apoptosis↓	[63]
	Aspirin	Rat NP cells (LPS)	ROS↓, inflammatory↓, catabolism↓	[106]
	Dexmedetomidine	Mice NP cells (IL-1*β*)	ROS↓, inflammatory↓	[107]

Biomaterial	ROS-SSR	Rats (needle puncture)	ROS↓, inflammatory↓	[108]
	Nanofullerol	Human NP cells (H_2_O_2_)	ROS↓, survival of cells↑, catabolism↓	[110]
	Fullerol nanoparticles	Mice BMSC (IL-1*β*)	ROS↓, inflammatory↓, Adipogenesis↓, catabolism↓	[111]
	KAM	Human ADSC (H_2_O_2_)	ROS↓, cell viability↑, inflammatory↓, catabolism↓, autophagy↑	[112]

Stem cell therapy	MSC-derived exosomes	Mice BMSC (H_2_O_2_)	ROS↓, inflammatory↓, mitochondrial dysfunction↓	[51]
	MSC-derived exosomes	Rat NP cells(compression)	ROS↓, apoptosis↓, mitochondrial dysfunction↓	[114]
	CESC-derived exosomes	Rat NP cells (TBHP)	Apoptosis↓, autophagy↑	[115]

NP: nucleus pulposus; AF: annulus fibrosus; ROS: reactive oxygen species; TBHP: tert-butyl hydroperoxide; ER: endoplasmic reticulum; NP-MSC: nucleus pulposus mesenchymal stem cell; LPS: lipopolysaccharides; ROS-SSR: ROS-scavenging scaffold with rapamycin; KAM: kartogenin and apocynin-loaded micelle; BMSC: bone marrow stromal cells; ADSC: adipose-derived stem cells; CESC: cartilage endplate stem cell.
